# Radiation Damage in XFEL: Case study from the oxygen-evolving complex of Photosystem II

**DOI:** 10.1038/srep36492

**Published:** 2016-11-09

**Authors:** Muhamed Amin, Ashraf Badawi, S. S. Obayya

**Affiliations:** 1 Department of Physics, City College of New York, New York, New York 10031, United States; 2 Center for Photonics and Smart Materials, Zewail City of Science and Technology, Sheikh Zayed District, 6th of October City, 12588 Giza, Egypt

## Abstract

Structural changes induced by radiation damage in X-ray crystallography hinder the ability to understand the structure/function relationship in chemical reactions. Serial femtosecond crystallography overcomes this problem by exposing the sample to very short and intense laser pulse leading to measurement before destruction. Here we use molecular modeling to map the radiation damage during the 10–50 fs to the intensity, the energy and the time duration of the laser pulse on the oxygen-evolving complex (OEC) of photosystem II. In the model, the nuclei move classically in a fully quantum potential created by electron density under the effect of strong laser pulse in the Ehrenfest dynamics regime. The results show that the Mn-Mn and Mn-Ca distances are less affected by radiation damage due to the their heavy masses, while one μ-oxo bridge (O5) moves significantly. The radiation damage may induce conformational changes of the water ligands but only bond elongation for the amino acids ligands. These effects are relatively intensity independent from 10^16^ to 10^17 ^W/cm^2^, but changes increase dramatically if the beam intensity is increased to 10^18^ W/cm^2^. In addition, the self amplified spontaneous emission (SASE) nature of the laser beam does not affect the dynamics of the ions.

In serial femtosecond crystallography randomly oriented crystals are exposed to very intense, ultra short laser pulses that produce one diffraction pattern in the tens of femtoseconds before it destroys the crystal^1^. One of the main advantages of this technique is that it is proposed to overcome the problem of radiation damage, which is caused by the interactions between the X-ray photons and the atoms in the sample. These interactions are statistical events dominated by the photoelectric effect^2^. Thus, it has been assumed that the atoms have little time to move during the brief time of the measurement.

The radiation damage induced by X-ray photons was studied as a stochastic process in which the probability of the photoelectric effect and inelastic events are measured as a function of the interaction cross section^2^. However, some quantum effects such as femtosecond collisional electron transfer and trapping of electrons due to the accumulated positive charges in the system were ignored. Because the geometrical structures of the atoms cannot change as fast as the electronic structure, Neutze and coworkers concluded that the obtained structures using this method may be considered radiation damage free structures^2^.

The structure of the oxygen-evolving center (OEC) of Photosystem II (PSII) that catalyzes the water splitting reaction has been studied intensively^3^,^4^,^5^,^6^,^7^,^8^,^9^. The OEC uses light energy to photo-oxidize water in a series of oxidation steps in the catalytic S-state cycle^10^. The first crystal structure of PSII at 3.1 Å resolution^3^ revealed that the OEC contains four Mn atoms, in addition to calcium atom. Due to the limited resolution the electron densities of the bridging oxygens that connect the Mn centers were not resolved. However, combining the lower resolution structural information with other data and computational studies provided good working models of the basic OEC structure^11^,^12^,^13^,^14^,^15^. The lowest catalytically active OEC (in the S_0_ state) has 3 Mn(III) and 1 Mn(IV).

However, the reported Mn-Mn distances in the 3.1 Å structure were not fully consistence with those found via Extended X-ray Absorption Fine Structure (EXAFS) spectroscopy and the differences were attributed to the radiation damage, which reduced the Mn in the OEC distorting the cluster^16^. In 2011, Umena and coworkers solved the PSII structure at 1.90 Å resolution and five bridging oxygens and all Mn ligands were identified^8^. However, the Mn-O distances remained longer than the bond distances measured by EXAFS or calculated using ab-initio calculations, which suggested that the Mn centers are reduced because of the free electrons generated due to the radiation damage^17^.

Serial femtosecond crystallography has been employed in the hope of obtaining a structure of the OEC free of radiation damage^18^,^19^. However, due to the limited resolution, the detailed structure of the OEC could not be resolved. Recently, Suga and coworkers obtained a radiation-damage-free structure by collecting still diffraction images of highly isomorphous crystals using the 10 KeV XFEL beam at a cryogenic temperature^20^.

Here, we study the radiation damage that might be expected in the intense XFEL beam using computer simulation. First, we optimized the geometry obtained from the crystal structure using localized orbital Density Functional Theory (DFT)^21^. Then, we applied an intense 10 KeV laser pulse for 50 femtoseconds and used Time Dependent Density Functional Theory (TDDFT) for the electrons to generate “on-the fly” potential that is applied on the classically moving ions in Ehrenfest Dynamics framework^22^,^23^. In addition, we have added absorption boundaries using a mask wavefunction to allow electrons to leave the system as will occur in the real reaction.

The deviation of each atom coordinates from the starting ground state position is evaluated by calculating the root mean square deviation (RMSD) for each atom type at each 0.15 femtosecond to map the radiation damage to the pulse duration. In addition, we study the evolution of the ion’s kinetic and potential energy, the total electronic energy and the total number of electrons lost through the exposure to the laser pulse.

Furthermore, we study how the beam intensity and energy correlate with the evolution of the RMSD of the nuclei and the energy of the system by applying laser pulses at different intensities and energies for 10 femtoseconds. Then we apply a combination of laser pulses that span a frequency band of 40 eV at different intensities to mimic the self-amplified spontaneous emission (SASE) spectrum and study its effect on the behavior of the system.

## Results and Discussions

In the “diffraction before destruction” XFEL experiment, an intense, ultra short laser pulse is applied to a sample, which produces a diffraction pattern recorded on the detector. The intense high-energy laser beam destroys the sample as it causes the electrons to be ejected from their orbitals and leave the system. The strong electrostatic repulsion between newly created ionic atoms stimulates them to move further from each other. The open question is; at which point of the destruction reaction is the diffraction pattern recorded?

### The evolution of the structure in time under the effect of laser field

A laser flash interacts with the PSII crystals in a nonadiabatic electronic process, which cannot be described in the Born-Oppenheimer regime. Ehrenfest dynamics can be used to model nonadiabatic interactions given that the time step is on the scale of the motion of electrons, which is order of magnitude faster than the nuclei. Thus, Ehrenfest dynamics is ideal for studying the effect of the short laser field on the OEC for times on the order of 50 fs. However, it should be noted that this method cannot describe the process in which, excited electrons dissipate energy into ionic vibrations known as Joule heating^24^.

**Figure 1:**
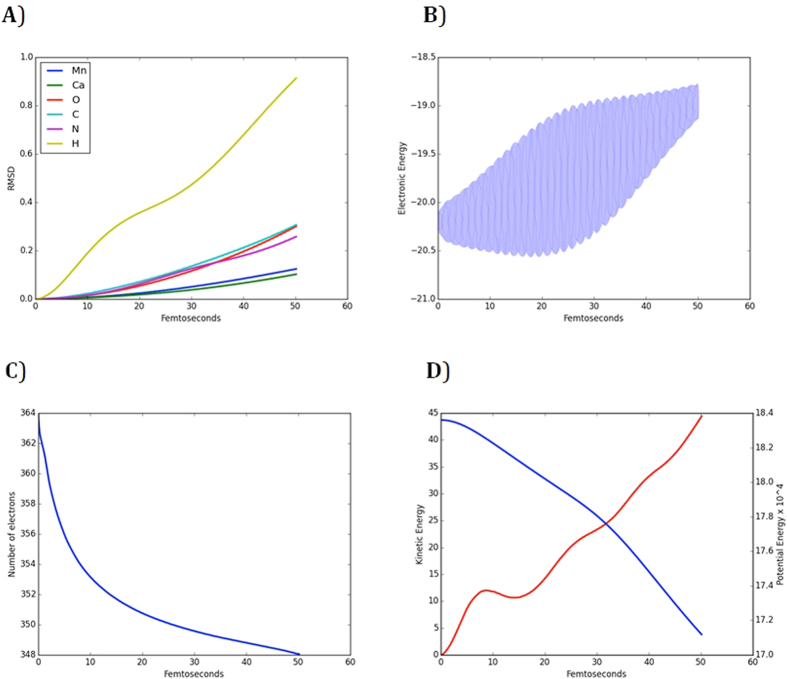
(**A**) The RMSD of the ions from their initial position at t = 0. (**B**) The evolution of electronic energies. (**C**) The evolution of the total electronic charges. (**D**) The evolution of the kinetic and potential energies of the ions through time.

The optimized X-ray structure is exposed to 50 fs laser pulse of 1.2 × 10^16^ W/cm^2^ intensity and 10 KeV photon energy; then the system is propagated in time using Approximated Enforced Time-Reversal Symmetry (AETRS) operator^25^ with a time step of 0.0015 fs (see supplementary information for simulation movie). The calculated RMSD from the initial position at t = 0 shows that the atoms are clustered in three groups (Fig. 1A); group 1 contains the hydrogen atoms, which have high kinetic energy due to their light mass; group 2 contains carbon, nitrogen and oxygen atoms where these atoms have similar masses and moves much slower than hydrogens; group 3 which contains manganese and calcium ions, which are the slowest moving ions due to their heavy masses. Thus, the Mn-Mn and Mn-Ca distances are expected to be more accurate and less affected by radiation damage.

The ion motion here is driven by a Coulomb explosion, that is to say as high energy photons are absorbed by the electrons, they gain enough energy to escape from their hosting atoms (Fig. 1B). The electrons disappear when they reach the boundary of the simulation box and the number of electrons in the system decreases rapidly in the first 5 fs. Then, the accumulated positive charges pose a potential barrier keeping the electrons from leaving the system (Fig. 1C). However, because the system is positively charged, the individual ions repel each other. Thus, the Coulomb electrostatic potential energy is converted to kinetic energy that induces the ionic motion (Fig. 1D).

**Figure 2:**
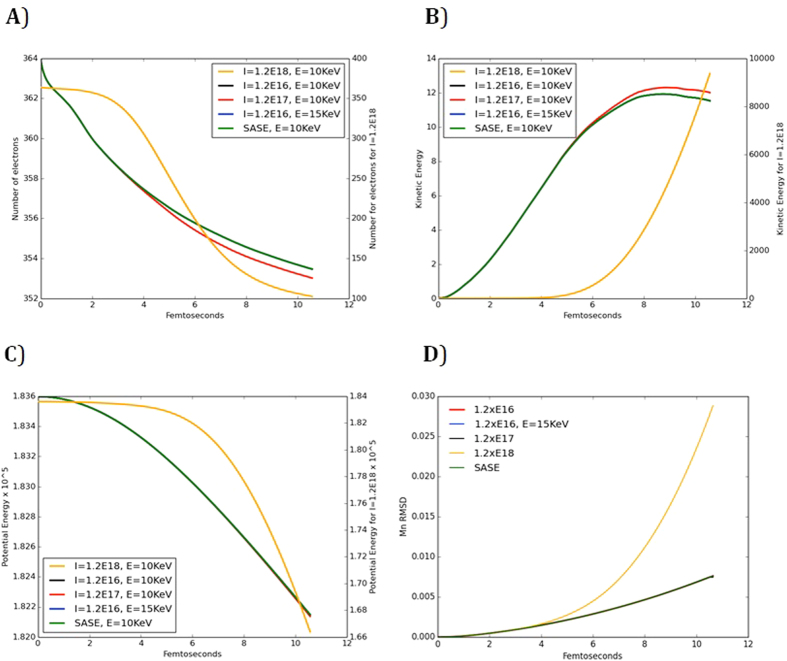
(**A**) The evolution of the total electronic charges. (**B**) The evolution of the kinetics energies of the ions through time. (**C**) The evolution of the potential energies of the ions. (**D**) The RMSD of the Mn ions from their initial position at t = 0.

To study the effect of the intensity of the laser field on the reaction, we applied laser pulses of intensities 1.2 × 10^16^, 1.2 × 10^17^ and 1.2 × 10^18 ^W/cm^2^ in three independent runs at energy of 10 KeV. The runs with intensities 10^16^ and 10^17^ W/cm^2^ yielded very similar results as shown in Fig. 2. However, if the intensity increased to 10^18^ W/cm^2^, the number of electrons that leave the system increases dramatically (Fig. 2A), which in turn increases the kinetic energy (and decreases the potential energy) of the ions (Fig. 2B,C) and causes severe radiation damage in very short time even for the heaviest ions (Fig. 2D). In contrast, increasing the energy of the beam to 15 KeV with the intensity fixed at 1.2 × 10^16^ for 10 fs does not produce any significant change in the RMSD of the ions or in the number of electrons lost.

**Figure 3:**
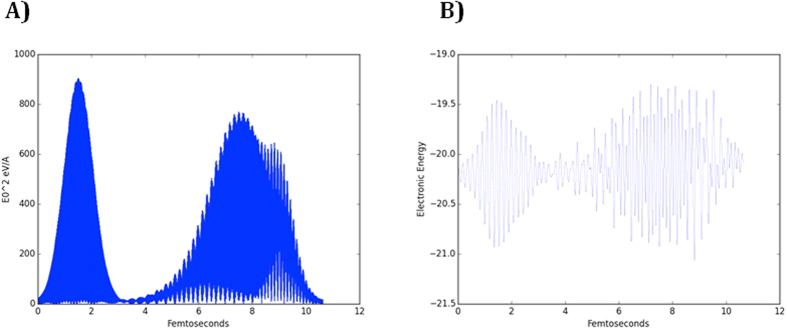
(**A**) The intensity profile of the applied laser pulse represented as the square of the amplitude of the electric field. The intensity is a superposition of 5 Gaussian pulses. The energies of the pulses are 10, 9.98, 9.993, 10.02 and 10.008 KeV. The intensities of the pulses are 1.2, 0.97, 0.01, 0.43 and 0.11 × 10^16^ W/cm^2^ respectively. (**B**) The evolution of electronic energy of the system in time.

The X-ray laser is often generated by Self-Amplified Spontaneous Emission, where free electrons are the lasing source. Due to the stochastic nature of the lasing electrons, the generated beam is not monochromatic and has a bandwidth of approximately 40 eV. Here, we model the effect of the SASE spectrum by applying 5 laser pulses of different frequencies and intensities simultaneously (Fig. 3A). The five pulses spans an energy range of 40 eV and the peak intensity is 1.2 × 10^16^ W/cm^2^. The results show that the SASE spectrum yields ion behavior that is identical to the case where a smooth Gaussian shape pulse with the same peak intensity is applied. The only observed difference is the temporal behavior of the electronic energy, which is driven by the applied spectrum (Fig. 3B).

### Insights on the radiation damage free structure of the OEC

The XFEL radiation damage free structure was obtained by applying a 10 fs laser pulse on highly isomorphous crystals^20^. The reported bond lengths differ significantly from the structure obtained from the traditional X-ray crystallography^8^ (Table 1a). These differences were attributed to the bond elongation resulting from reducing the Mn centers induced by radiation damage.

The geometry optimization of the X-ray or XFEL structures using DFT leads to structures with good agreement with EXAFS^17^,^26^. However, the calculated Mn-O5 distances do not match the measured values (Table 1b DFT column). To test if these structural changes are resulting from the radiation damage, we expose the DFT optimized structure to a laser pulse of 10 KeV energy and 1.2 × 10^16^ W/cm^2^ intensity for 10 fs (Table 1b 10 fs column). The elongated bonds due to the exposure to the laser filed are slightly in better agreement with XFEL measurements. However, the Mn4-O5 and Mn3-O5 distances are 0.40 and 0.28 Å shorter than the measured bond lengths respectively. Batista and coworkers suggested the structure is a mixture of the S_0_ and S_1_ states due to the dark adaptation prior to data collection^26^.

To further examine the structure, we exposed the DFT structure to a laser pulse of 50 fs to check if the structure will converge at any point to the XFEL structure (Table 1b 50 fs column). Although the Mn-O5 and Ca-O5 distances agree better with the XFEL structure, the Mn-Mn distances are longer and diverging from the measured bond lengths. Thus, our simulation suggests that the Mn-O5 bonds elongation in the XFEL structure is not caused by radiation damage.

## Methods

### TDDFT and Ehrenfest dynamics

Starting from the DFT optimized structure in the localized orbital framework; the ground state wavefunction is obtained in the real space grid representation in OCTOPUS^27^,^28^ with 0.18 Å grid spacing. Then, the time propagation method is used to evolve the ground state wavefunction in time with 0.0015 fs time step in the existence of Gaussian shape laser field of 1.2 × 10^16^, 1.2 × 10^17^ or 1.2 × 10^18^ W/cm^2^. In addition, a mask wavefunction is applied on the borders of the simulation box to absorb the electrons reaching there to mimic the real reaction^27^,^28^. The ions are allowed to move in Ehrenfest dynamics framework. The beam energy operated on PSII crystals at Stanford Linear Accelerator Center (SLAC) is less than 10 KeV^19^,^29^. However, to study the extreme case the input laser energy in our calculations was set to 10 KeV and 15 KeV to study the effect of energy increase.

### Geometry optimization

**Figure 4:**
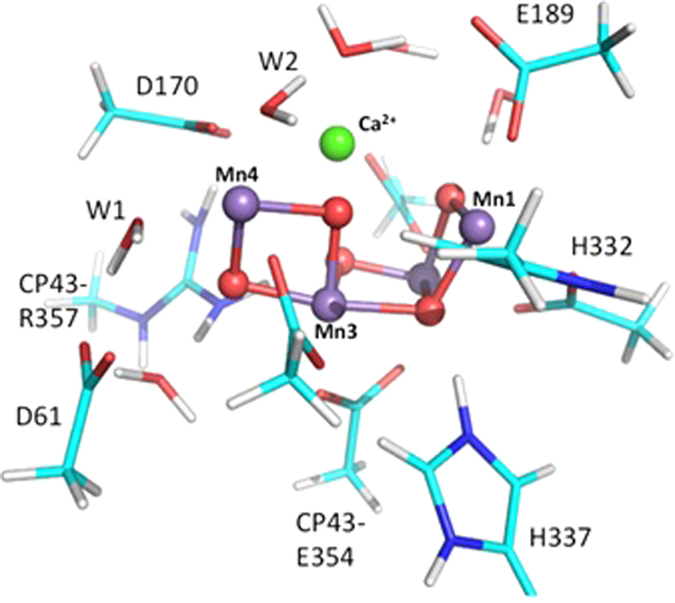
The DFT optimized structure starting from the X-ray structure (PDB code: 3ARC). The model includes 115 atoms. The Mn_4_O_5_Ca^2+^ cluster is shown in spheres.

Because OCTOPUS^27^,^28^ is not designed for geometry optimization, the structure is optimized with localized orbitals DFT using the B3LYP density functional in Gaussian09^21^ starting from the 1.9 Å structure of PSII (PDB ID **3ARC**)^8^. The LANL2DZ basis sets with effective core potentials are used for Mn and Ca, while 6-31G* is used for the other atoms. The model includes the four terminal waters bound to Mn4 and Ca^2+^, the side chains of the amino-acid ligands to each Mn (D170, E189, H332, E333, D342, A344, and CP43-E354), as well as the side chains hydrogen bonded to the bridging and terminal oxygens (D61, H337, and CP43-R357). In addition, 5 crystallographic waters are included in the model (Fig. 4). All Mn centers are fixed in the high spin states.

## Additional Information

**How to cite this article**: Amin, M. *et al*. Radiation Damage in XFEL: Case study from the oxygen-evolving complex of Photosystem II. *Sci. Rep*. **6**, 36492; doi: 10.1038/srep36492 (2016).

## Supplementary Material

Supplementary Information

Supplementary Video S1

## Figures and Tables

**Table 1:** The bond length of X-ray, XFEL, DFT after 10 fs and after 50 fs ab-initio molecular dynamics simulation.

	a) Measurements		b) Simulation		
	X-ray	XFEL	DFT	10 fs	50 fs
Mn4-O5	2.5	2.32	1.88	1.92	2.34
Mn3-O5	**2.4**	**2.17**	**1.87**	**1.89**	**2.01**
Ca-O5	2.7	2.59	2.47	2.47	2.53
Mn4-Mn3	3.0	2.86	2.80	2.81	2.94
Mn2-Mn3	2.9	2.70	2.89	2.90	3.08
Mn1-Mn2	2.8	2.67	2.88	2.88	3.06
Mn1-Mn3	3.3	3.24	3.20	3.20	3.34

All distances are measured in Angstrom. The bold values have a large deviation from the XFEL structure. X-ray^8^ represents the distances measured based on the synchrotron source at 1.90 Å and XFEL^20^ for the free electron laser source at 1.95 Å.
