# Supplementary material for: Radiation Damage in XFEL: Case study from the oxygen-evolving complex of Photosystem II
Source: Sci Rep. 2016 Nov 9;6:36492. doi: 10.1038/srep36492 (PMC5101503; doi:10.1038/srep36492)
Supplement: Supplementary Information [file srep36492-s1.pdf]

Supplementary Information for

Radiation Damage in XFEL: Case study from the oxygen-evolving complex of Photosystem II

Muhamed Amin[1,2*], Ashraf Badawi[2], S. S. Obayya[2]

[1] Department of Physics, City College of New York, New York, New York 10031, United States

[2] Center for Photonics and Smart Materials, Zewail City of Science and Technology, Sheikh Zayed District, 6th of October City, 12588 Giza, Egypt

Correspondence to mamin@ccny.cuny.edu.

Description of Radiation damage movie. The movie is constructed by merging 221 frames that represent the evolved structures every 0.2 femtoseconds. The total simulation time is 50fs. Through the simulation, the structure is exposed to Gaussian laser pulse of 50fs length, 10KeV energy and $1.2x10^{16}$ W/cm^2 intensity. The Mn ions are shown in magenta, oxygens in red and calcium in green.
